# mTORC2 promotes pancreatic cancer progression and parp inhibitor resistance

**DOI:** 10.32604/or.2023.029309

**Published:** 2023-06-27

**Authors:** CHIWEN BU, LIGANG ZHAO, LISHAN WANG, ZEQIAN YU, JIAHUA ZHOU

**Affiliations:** 1 Department of Hepatobiliary and Pancreatic Surgery, Zhongda Hospital, School of Medicine, Southeast University, Nanjing, 210009, China; 2 School of Medicine, Southeast University, Nanjing, 210009, China

**Keywords:** mTORC2, Pancreatic cancer, PARP inhibitors, HR repair, DNA damage

## Abstract

Pancreatic cancer is one of the most aggressive cancers with a median survival time of less than 5 months, and conventional chemotherapeutics are the main treatment strategy. Poly(ADP-ribose) polymerase (PARP) inhibitors have been recently approved for BRCA1/2-mutant pancreatic cancer, opening a new era for targeted therapy for this disease. However, most pancreatic cancer patients carry wild-type BRCA1/2 with resistance to PARP inhibitors. Here, we reported that mammalian target of rapamycin complex 2 (mTORC2) kinase is overexpressed in pancreatic cancer tissues and promotes pancreatic cancer cell growth and invasion. Moreover, we found that knockdown of the mTORC2 obligate subunit Rictor sensitized pancreatic cancer cells to the PARP inhibitor olaparib. Mechanistically, we showed that mTORC2 positively regulates homologous recombination (HR) repair by modulating BRCA1 recruitment to DNA double-strand breaks (DSBs). In addition, we confirmed that combination treatment with the mTORC2 inhibitor PP242 and the PARP inhibitor olaparib synergistically inhibited pancreatic cancer growth *in vivo*. Thus, this study provides a novel target and strategy for optimizing PARP inhibitor efficiency in pancreatic cancers.

## Introduction

Pancreatic ductal adenocarcinoma (PDAC) accounts for approximately 85% of all cancer cases derived from the pancreas [1]. Although PDAC is not a common solid cancer and represents only approximately 3% of newly diagnosed cancer cases each year, it has been considered the leading cause of cancer-related death within the last 10 years [2]. Due to its features of rapid progression and radiochemoresistance, the five-year survival rate of PDAC is less than 15% [3]. Currently, paclitaxel and gemcitabine-based chemotherapy is the standard of care for PDAC treatment, and there is no effective targeted therapeutic approach against PDAC [4–6]. Recently, poly (ADP-ribose) polymerase (PARP) inhibitors, including olaparib, have been approved for maintenance treatment of PDAC patients diagnosed with germline BRCA1/2 gene mutations [7,8], which opens a new era for targeted therapy for PDAC. BRCA1 mutations are detected in approximately 2.4% of PDAC cases, and 5.7% of PDACs harbor BRCA2 mutations [9]. Thus, the majority of PDAC patients carry wild-type (WT) BRCA1/2 and are not eligible for PARP inhibitor treatment. Therefore, designing a strategy that confers a “BRCAness” phenotype in PDAC cancer cells might render PDAC patients with WT BRCA1/2 susceptible to PARP inhibition.

PARPs are a class of enzymes that catalyze the poly ADP-ribosylation (PARylation) of substrate proteins and are involved in various cellular functions, including DNA repair, metabolism, and cell death [10–12]. The PARP family consists of 17 members, of which PARP1 is the most studied [13]. PARP proteins participate in DNA single-strand break (SSB) repair, and inhibition of PARP results in accumulation of unrepairable DNA SSBs and the subsequent formation of lethal DNA double-strand breaks (DSBs); the fate of cells with DNA DSBs is highly dependent on homologous recombination (HR)-mediated DNA repair [14]. BRCA1 and BRCA2 proteins are well-known HR-mediated repair regulators, and loss of BRCA1/2 function leads to compromised HR-mediated repair capacity in cancer cells [15], so cells presenting this phenotype are highly sensitive to PARP inhibitor treatment. This synthetic lethality between PARP inhibitors and HR-mediated repair deficiency (HRD) indicates that combining HR-mediated repair inhibitors and PARP inhibitors could be a promising cancer treatment strategy.

Mammalian target of rapamycin (mTOR) kinase, a phosphoinositide 3-kinase-related protein kinase, functions in two different protein complexes termed mTOR complex 1 (mTORC1) and mTOR complex 2 (mTORC2) [16]. mTORC1 consists of mTOR, Raptor, MLST8, PRAS40 and DEPTOR, while mTORC2 consists of mTOR, Rictor, SIN, and GβL [17]. As the rapamycin-sensitive kinase complex, the function of mTORC1 has been widely investigated, while the roles of the rapamycin-insensitive mTORC2 complex remain unclear. Recently, accumulating evidence has revealed that mTORC2 is hyperactivated in lung cancer tissues and is a favorable prognostic factor for lung cancer [18]. However, the functions of mTORC2 in PDAC are largely unknown. Here, we showed that mTORC2 plays oncogenic roles in PDAC cell growth and invasion. More importantly, we report here that mTORC2 promotes HR-mediated repair by regulating the DNA repair protein BRCA1, which consequently confers PARP inhibitor resistance to PDAC cells. Thus, targeting mTORC2 sensitizes PDAC cells to PARP inhibitor treatment *in vitro* and *in vivo*. Therefore, our present study provides a novel therapeutic target to optimize PARP inhibitor therapy in BRCA1/2 WT PDAC patients.

## Materials and Methods

### Cell culture and transfection

PANC-1, MIA PaCa-2 and HEK293FT cells were obtained from ATCC (American Type Culture Collection) and cultured in Dulbecco’s modified Eagle’s medium supplemented with 10% fetal bovine serum (FBS, ExCell Bio, Shanghai, China) and 100 U/mL penicillin/streptomycin (#15140122, ThermoFisher Scientific, Waltham, MA, USA). Cell transfection with DNA or siRNA was performed using Lipofectamine 3000 (#L3000001, Thermo Fisher Scientific, Waltham, MA, USA) according to the manufacturer’s instructions.

### Antibodies and reagents

Anti-BRCA1 (#14823), anti-Ki67 (#9449), Alexa Fluor® 488 (#8878) and Alexa Fluor® 555 (#8953) antibodies were purchased from Cell Signaling Technology (Danvers, MA). Anti-Rictor antibody (A300-459A) was purchased from Bethyl Laboratories (Montgomery, USA). Anti-γH2AX (05-636) antibody and anti-Rad51 (PC-130) antibody were obtained from EMD Millipore (Norwood, USA). Anti-β-actin antibody was purchased from Santa Cruz Biotechnology (Santa Cruz, CA). pDR-GFP (#17617), pCBASceI (#26477) and Rictor shRNA plasmids (#1853 and #1854) were obtained from Addgene (Watertown, MA, USA). Olaparib was purchased from Selleckchem (Houston, TX).

### Lentivirus construction and Rictor knockdown stable cell line generation

Rictor shRNA was purchased from Addgene (#1853 and #1854, Watertown, MA, USA), and the sequences were as follows: Rictor shRNA1, 5′-CCG GTA CTT GTG AAG AAT CGT ATC TTC TCG AGA AGA TAC GAT TCT TCA CA A GTT TT TTG-3′; Rictor shRNA2, 5′-CGG GCA GCC TTG AAC TGT TTA ACT TCC TGT CAT TAA ACA GTT CAA GGC TGC TTT TTG AAT T-3′. Rictor shRNA plasmids were cotransfected with packing plasmids (psPAX2 and pCMV-VSV-G) into HEK293FT cells. Seventy-two hours after transfection, the supernatant containing the lentivirus particles was collected and added to the cells along with polybrene (10 μg/ml) (#sc-134220 Santa Cruz Biotechnology, Santa Cruz, CA) to generate the Rictor-silenced stable cell line. Twenty-four hours after lentivirus infection, cells were selected in the presence of 1–3 μg/ml puromycin (#A610593, Sangon Biotech, Shanghai, China). The stable clones were then picked, and Rictor expression was validated by western blotting.

### Immunohistochemical (IHC) staining

Paraffin-embedded tumor tissues were cut into 5-μM-thick sections, and IHC staining was performed using RTU Vectastain Kit (Vector Laboratories, Newark, CA, USA) according to the manufacturer’s instructions. Briefly, the sections were deparaffined in xylene and rehydrated in graded ethanol. After inactivation of endogenous peroxidase by H2O2 and antigen retrieval, slides were blocked in 5% goat serum and incubated with primary antibody including anti-Rictor (A300-459A, Bethyl Laboratories, 1:500), anti-Ki67(#9449, Cell signaling technology, 1:1000) and anti-γH2AX(05-636, EMD Millipore, 1:600) at 4°C overnight. Then, the slides were incubated with pan-anti-mouse/rabbit/goat secondary antibody (#BA-1300, included in the RTU Vectastain Kit) and visualized using 3,3-diaminobenzidine tetrahydrochloride (DAB) staining solution.

### Immunofluorescence (IF) staining

Cells were planted and grown on chamber slides. After treatment, the cells were washed with PBS, fixed with 4% paraformaldehyde and permeabilized with 0.3% Triton X-100. Then, slides were blocked with 10% goat serum and incubated with primary antibodies including anti-Rad51 (PC-130, EMD Millipore, 1:500) and anti-BRCA1 (sc-6954, Santa Cruz Biotechnology, 1:200) at 4°C overnight. Then, the slides were extensively washed with PBS and incubated with Alexa Fluor secondary antibodies (1:5000, Cell Signaling Technology) in the dark for 1 h. After washing with PBS and mounting with Prolong Gold antifade DAPI, the slides were assessed using a confocal microscope.

### Comet assay

The comet assay was performed using the Comet SCGE assay kit (Enzo, New York, USA) according to the manufacturer’s instructions. Briefly, the cell suspension was mixed with molten LMAgarose and added onto the Comet Slide. After the agarose solidified at 4°C, the slides were immersed in precooled lysis buffer at 4°C for 1 h. After incubation in alkaline solution (300 mM NaOH and 1 mM EDTA), the slides were washed in TBE buffer (89 mM Tris-boric and 2 mM EDTA), followed by electrophoresis and staining with CYGreen Dye (provided in the kit) for 30 min. Then, the images were collected using fluorescence microscopy, and the tail DNA percentage was quantified by OpenComet software.

### Homologous recombination (HR) repair reporter assay

U2OS-DR-GFP cells harboring the HR reporter cassette were infected with lentivirus carrying control (ctrl) or Rictor shRNA to silence Rictor expression. After selection with puromycin, cells were transfected with the pCBASceI plasmid to induce DNA DSBs, followed by flow cytometry analysis of GFP expression at 48 h after pCBASceI transfection. HR-mediated repair efficacy is positively correlated with the percentage of GFP-expressing cells.

### Pancretic cancer xenografts

The animal experiments were approved by the Institutional Animal Care and Use Committee of Southeast University. PANC-1 cells (1 × 10^6^) in 100 μl PBS were inoculated subcutaneously into 6-week-old female BALB/c nude mice (GemPharmatech Co., Ltd., Nanjing, China). Tumor-bearing mice were randomly grouped when the average tumor volume reached approximately 100 mm^3^. Mice were administered olaparib (orally, 100 mg/kg) (S1060, Selleck, Houston, USA), PP242 (intraperitoneal (IP) injection, 20 mg/kg) (S2218, Selleck, Houston, USA), or both. Tumor volume was measured every three days. At the endpoint of the experiment, the mice were sacrificed, and tumor tissues were collected and fixed in formalin.

### Statistical analysis

All data are shown from one representative experiment of at least three independent experiments and are presented as the mean ± standard deviation (SD). The statistical significance of differences between groups was analyzed with two-sided Student’s *t* test. The results were considered statistically significant at *p* < 0.05.

## Results

### mTORC2 obligate component Rictor is elevated in PDAC tissues and associated with a poor prognosis

**Figure 1:**
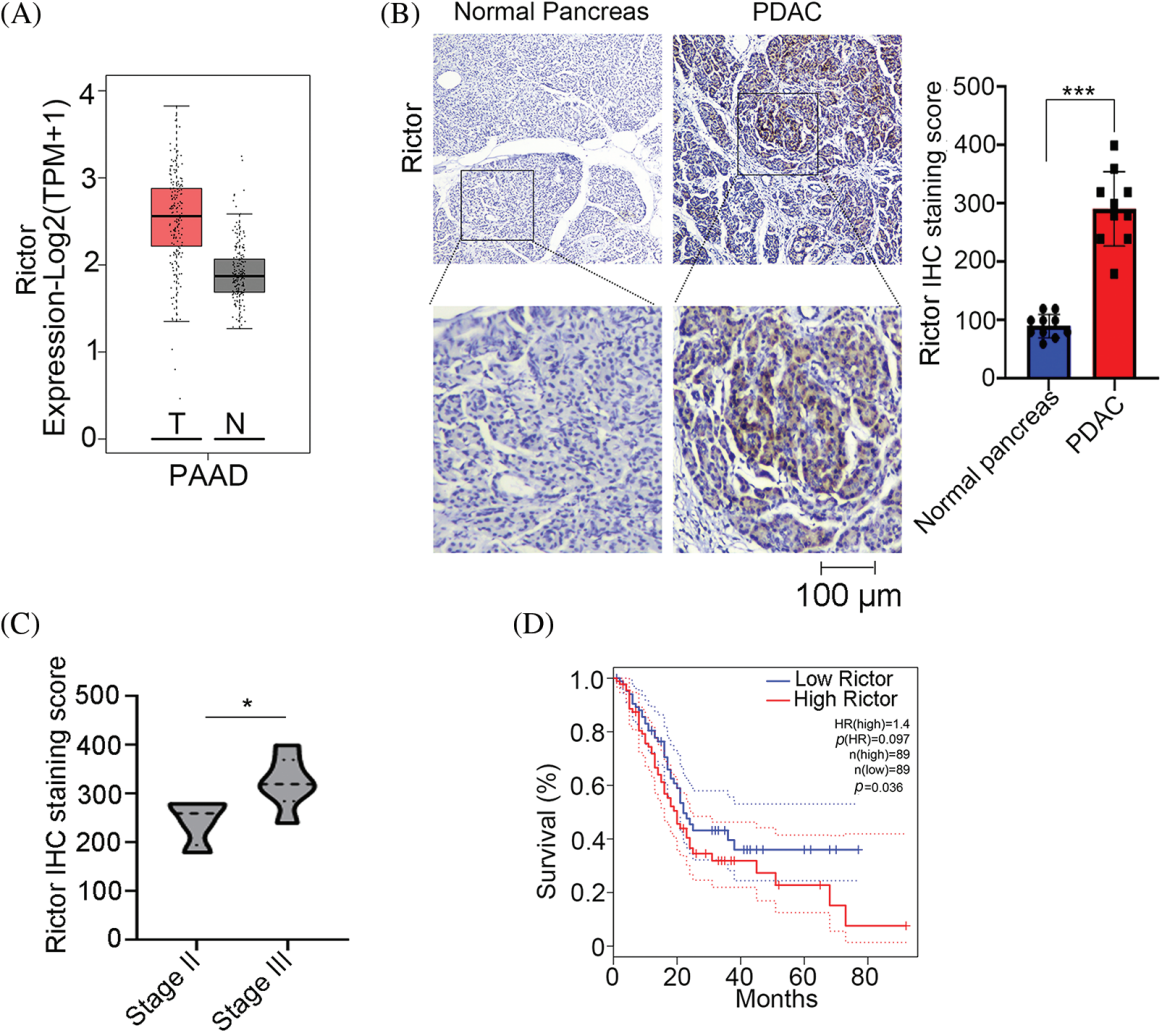
The mTORC2 obligate component Rictor is overexpressed and associated with poor survival in PDAC patients. (A) Analysis of Rictor mRNA levels in pancreatic adenocarcinoma (PAAD) tumor (T) tissues (n = 179) and normal pancreas (N) tissues (n = 171) through The Cancer Genome Atlas (TCGA) database. (B) IHC analysis of Rictor protein expression in pancreatic ductal adenocarcinoma (PDAC) tissues and normal pancreas tissues. Representative images of IHC staining (left) and quantification of the IHC score (right) are shown. Data are presented as the mean ± standard deviation (SD), n = 10. ***p < 0.001 according to 2-tailed *t* test. (C) Analysis of Rictor protein level in different stage of PDAC tumors. *p < 0.05 by 2-tailed *t* test. (D) Kaplan‒Meier survival analysis of Rictor expression in PDAC patients (n = 178) from the TCGA database.

Rictor is an mTORC2-specific subunit that is indispensable for mTORC2 function [18]. The expression level of Rictor is positively correlated with mTORC2 enzymatic activity [18]; thus, Rictor is considered a biomarker predicting mTORC2 activity. To explore the role of mTORC2 in pancreatic cancer, we first analyzed Rictor expression in pancreatic adenocarcinoma (PAAD) tumor (T) tissues and normal (N) pancreas tissues using mRNA expression data collected from The Cancer Genome Atlas (TCGA). As shown in Fig. 1A, a higher level of Rictor mRNA was detected in tumor tissues than in normal pancreatic tissues. Then, we performed immunohistochemical (IHC) analysis of Rictor protein expression in pancreatic ductal adenocarcinoma (PDAC) tissues and normal pancreas tissues. Consistently, we observed significantly higher Rictor protein levels in PDAC tissues than in normal pancreas tissues (Table 1 and Fig. 1B). And higher level of Rictor was detected in stage III tumors compared with stage II tumors (Fig. 1C). Moreover, Kaplan‒Meier survival analysis revealed that a high level of Rictor is associated with a poor prognosis in PDAC patients (Fig. 1D), suggesting that mTORC2 might act as an oncogenic factor in PDAC patients.

**Table 1:** Clinicopathological parameters

Clinical parameter	Status	n(%)
Gender	Male	7(70%)
	Female	3(30%)
Age	<60	4(40%)
	>60	6(60%)
Histopathological type	PDAC	10(100%)
Tumor stage	T2	4(40%)
	T3	6(60%)
Lymph nodes	N0	5(50%)
	N1	3(30%)
	N2	2(20%)
Metastasis	M0	10(100%)
	M1	0

### mTORC2 inhibition suppresses PDAC cell growth and invasion

**Figure 2:**
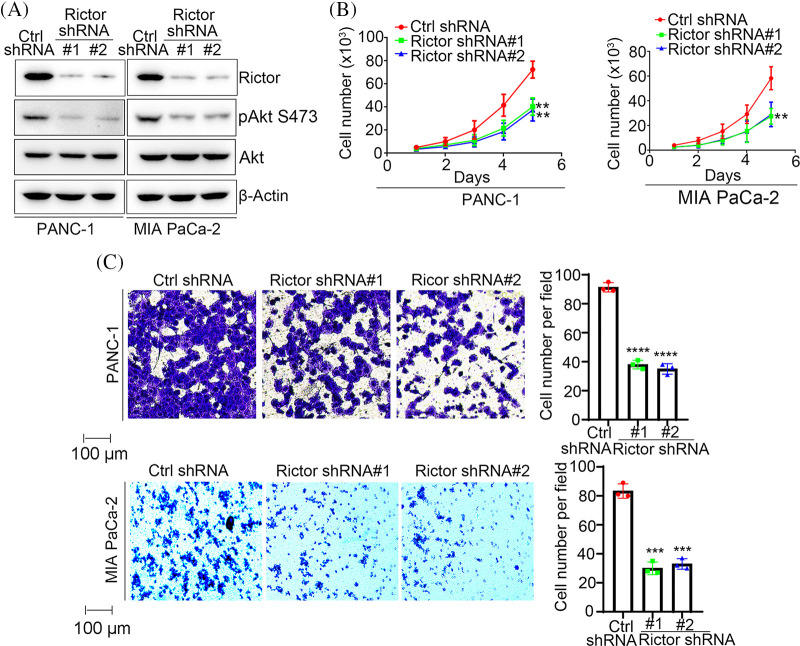
Knockdown of Rictor suppresses PDAC cell growth and invasion. (A) Western blot analysis of Rictor levels in PANC-1 or MIA PaCa-2 cells expressing Rictor shRNA. (B) Cell growth analysis of cells expressing Rictor shRNA. (C) Transwell invasion assay of ANC-1 or MIA PaCa-2 cells stably transfected with control (Ctrl) or Rictor shRNA. Representative images (left) and quantification (right) of invaded cells are shown. Data are presented as the mean ± standard deviation (SD) of three separate experiments, n = 3. **p < 0.01, ***p < 0.001, and ****p < 0.0001 according to 2-tailed *t* test.

We next silenced Rictor in two PDAC cell lines, PANC-1 and MIA PaCa-2, to further assess the oncogenic functions of mTORC2 in PDAC cells (Fig. 2A). Silence of Rictor significantly inhibited phosphorylation of Akt S473, which is a substrate of mTORC2 and is considered as a biomarker for mTORC2 activation (Fig. 2A). This result indicating knockdown of Rictor significantly impaired mTORC2 activity. As shown in Fig. 2B, we found that Rictor silencing slowed cell growth in both PANC-1 and MIA Paca-2 cells. Then, we performed a Matrigel-coated transwell invasion assay to assess whether mTORC2 affects PDAC invasion. As expected, we found that Rictor depletion significantly reduced the invasion capacity of both PANC-1 and MIA Paca-2 cells (Fig. 2C). These results indicate that mTORC2 positively regulates cancer cell growth and invasion in PDAC cells.

### mTORC2 inhibition sensitizes PDAC cells to PARP inhibitor treatment

**Figure 3:**
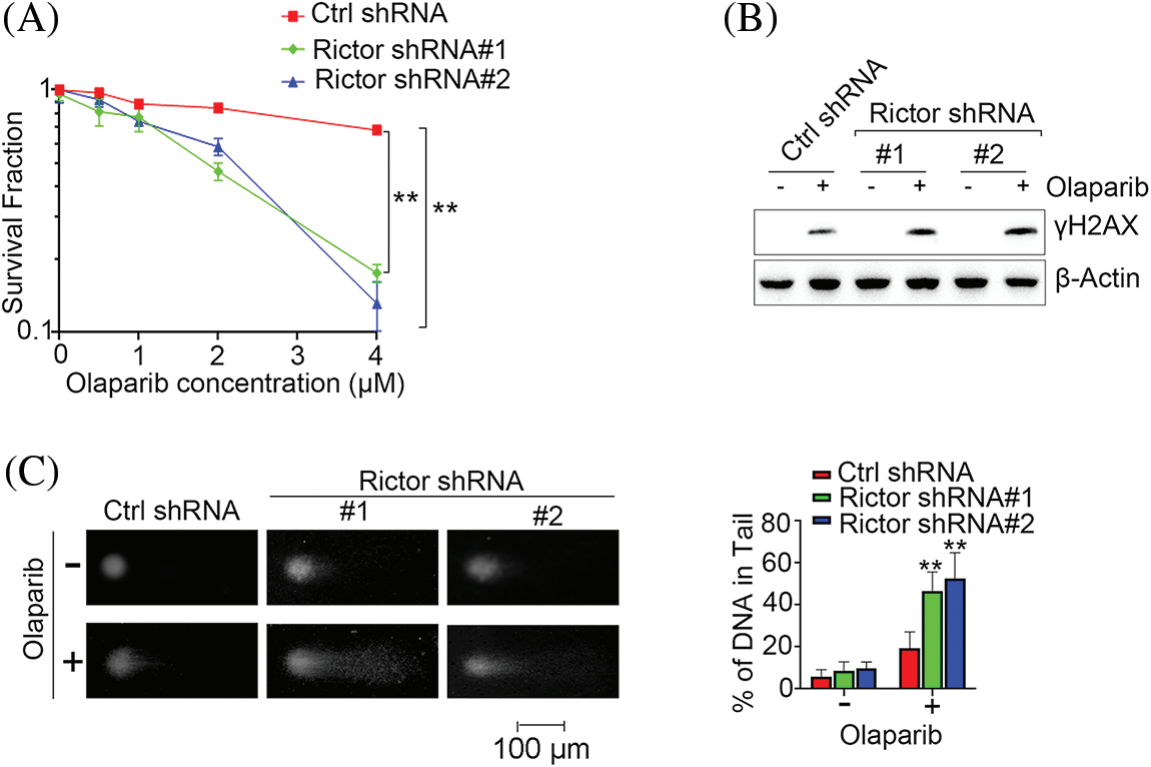
Rictor knockdown enhances DNA damage induced by the PARP inhibitor olaparib in PDAC cells. (A) PANC-1 cells expressing control (Ctrl) or Rictor shRNA were treated with the indicated concentrations of olaparib, followed by clonogenic survival analysis. (B, C) PANC-1 cells expressing control (Ctrl) or Rictor shRNA were treated with or without 20 μM olaparib for 24 h, followed by western blot analysis of γH2AX (B) and comet DNA damage assay (C). Data are presented as the mean ± standard deviation (SD) of three separate experiments, n = 3. **p < 0.01 according to 2-tailed *t* test.

PARP inhibitors, including olaparib, have been approved to treat PDAC patients and represent a novel therapeutic regimen for PDAC patients [8]. We then assessed whether mTORC2 deficiency could confer PARP inhibitor sensitivity in PDAC cells. Clonogenic survival assays showed that silencing Rictor significantly enhanced sensitivity to the PARP inhibitor olaparib (Fig. 3A). PARP inhibitors kill tumor cells primarily through the induction of DNA DSBs [19]. Thus, we evaluated olaparib-induced formation of γH2AX, which is H2AX phosphorylated at the S139 site and is recognized as the biomarker for DNA DSBs, in cells expressing control or Rictor shRNA. Consistent with the results of the clonogenic survival assay, we observed significantly higher levels of γH2AX in Rictor knockdown cells than in control cells (Fig. 3B). To confirm the increased DNA damage induced by olaparib in Rictor-knockdown cells, we then employed a comet assay, a gel electrophoresis-based assay that directly measures intracellular DNA damage, to further evaluate DNA damage after olaparib treatment. Similar to the previous results, we detected significantly more comet tails in cells expressing Rictor shRNA than in control cells (Fig. 3C), indicating that olaparib caused higher levels of DNA damage in Rictor knockdown cells than in control cells.

### mTORC2 positively regulates HR-mediated repair

**Figure 4:**
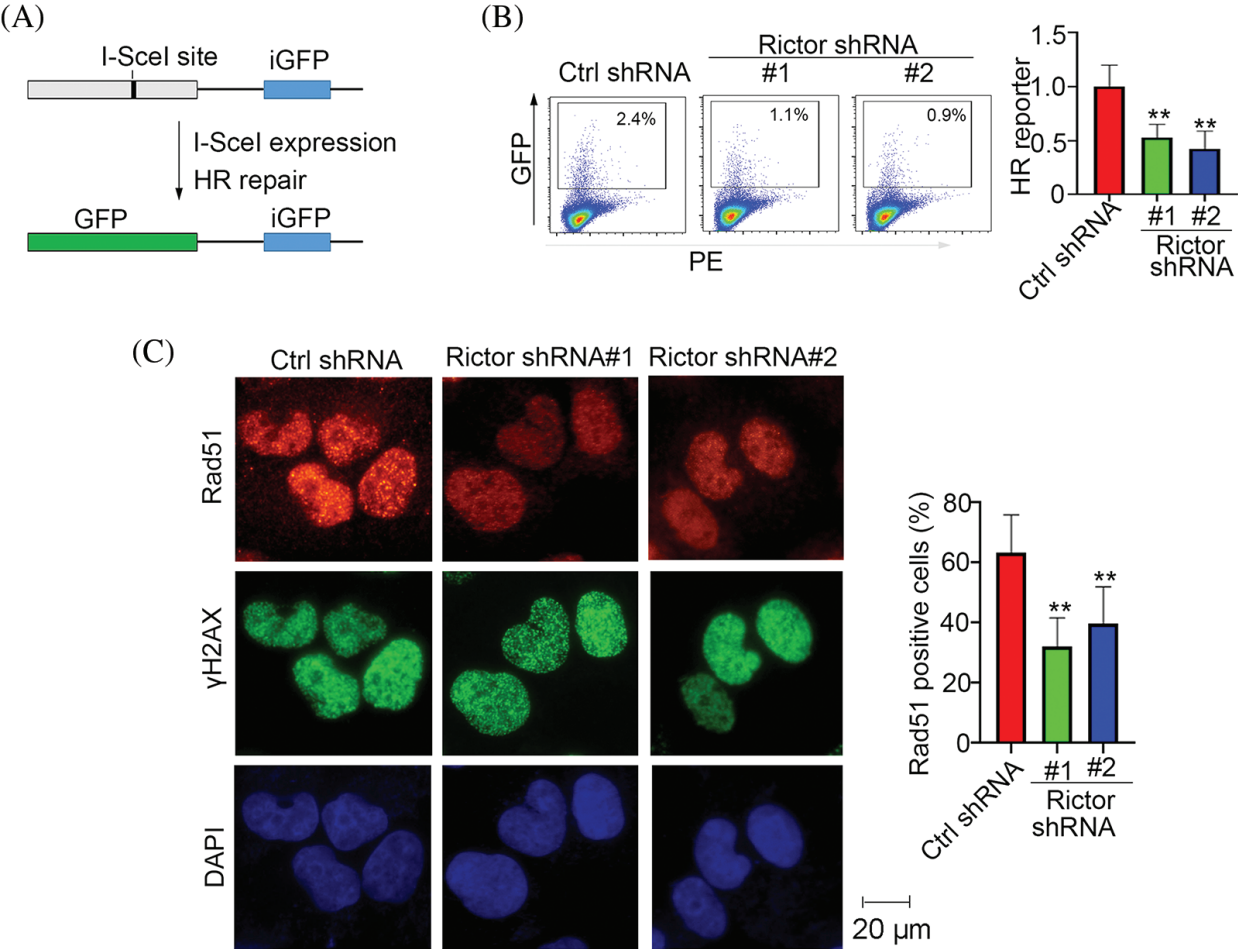
Knockdown of Rictor suppresses HR-mediated repair. (A) Diagram of HR-mediated repair reporter, in which a GFP gene is interrupted by insertion of endonuclease I-SceI cut site; the functional GFP can be restored only by HR-mediated repair using the downstream iGFP sequence as a template. (B) HR-mediated repair capacity was measured in U2OS-DR-GFP cells transfected with control (Ctrl) or Rictor shRNA. (C) PANC-1 cells expressing control (Ctrl) or Rictor shRNA were treated with 20 μM olaparib, followed by immunofluorescence analysis of Rad51 foci. Data are presented as the mean ± standard deviation (SD) of three separate experiments, n = 3. **p < 0.01 according to 2-tailed *t* test.

HR-mediated repair capacity is the primary factor determining the number of olaparib-generated DNA DSBs [8]. We then employed an HR-mediated repair reporter system in which a GFP gene was disrupted by an endonuclease I-SceI cutting site and the functional GFP sequence could only be restored by HR-mediated repair using the downstream iGFP template (Fig. 4A). By using this reporter, we found that silencing Rictor significantly reduced HR-mediated repair activity (Fig. 4B). Rad51 is a recombinase that catalyzes strand invasion and recombination during the HR-mediated repair process [20]. Rad51 foci formation at DNA DSBs is considered a biomarker of HR-mediated repair [20]. Consistent with the reporter assay, we detected a significant reduction in Rad51 foci number in cells expressing Rictor shRNA (Fig. 4C). These results demonstrate that loss of mTORC2 activity decreased the HR-mediated repair capacity in cells.

### mTORC2 promotes HR-mediated repair by regulating BRCA1

**Figure 5:**
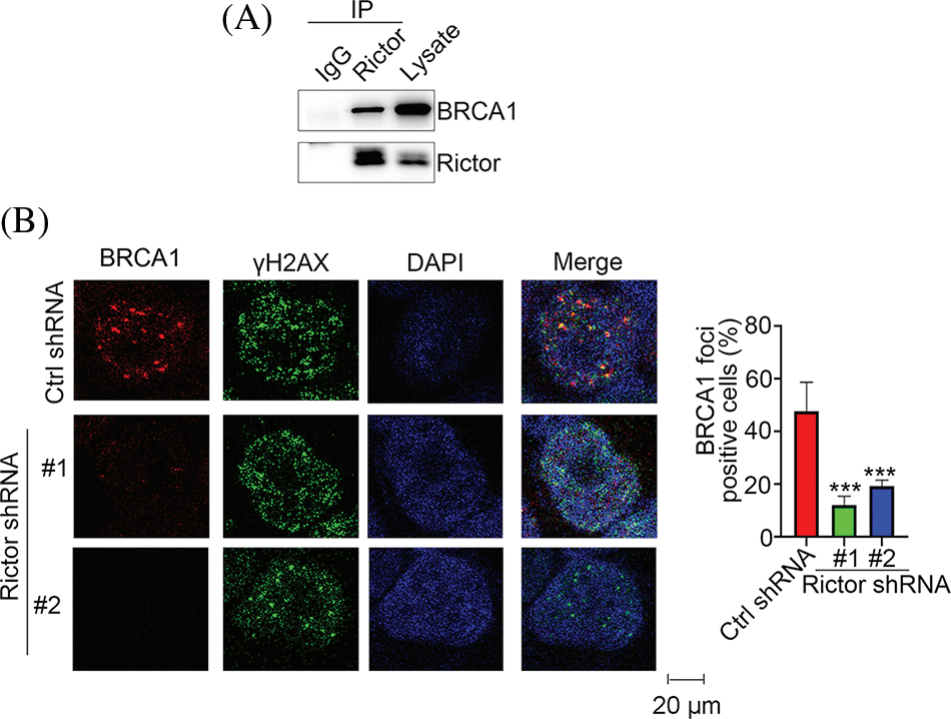
Rictor interacts with BRCA1. (A) Coimmunoprecipitation (Co-IP) analysis of the Rictor/BRCA1 association in PANC-1 cells using an anti-Rictor antibody. (B) PANC-1 cells expressing control (Ctrl) or Rictor shRNA were treated with 20 μM olaparib, followed by analysis of BRCA1 foci. Data are presented as the mean ± standard deviation (SD) of three separate experiments, n = 3. ***p < 0.001 according to 2-tailed *t* test.

To explore the underlying mechanism by which mTORC2 affects HR-mediated repair, we employed an immunoprecipitation (IP) assay and found that the mTORC2-specific protein Rictor is associated with BRCA1 (Fig. 5A). BRCA1 is a well-known tumor suppressive protein that is involved in the regulation of HR-mediated repair, cell cycle regulation and transcription [21]. BRCA1 can be recruited to the DNA DSB site and directly participate in the regulation of HR-mediated repair [21]. Thus, we then evaluated BRCA1 foci formation after olaparib treatment in PANC-1 cells expressing control or Rictor shRNA. Interestingly, we detected a significant decrease in BRCA1 foci number in Rictor-silenced cells compared with control cells (Fig. 5B). These results suggested that the decrease in HR-mediated repair capacity in Rictor knockdown cells resulted from the inhibition of BRCA1 function.

### Inhibition of mTORC2 sensitizes PDAC to PARP inhibitor treatment

**Figure 6:**
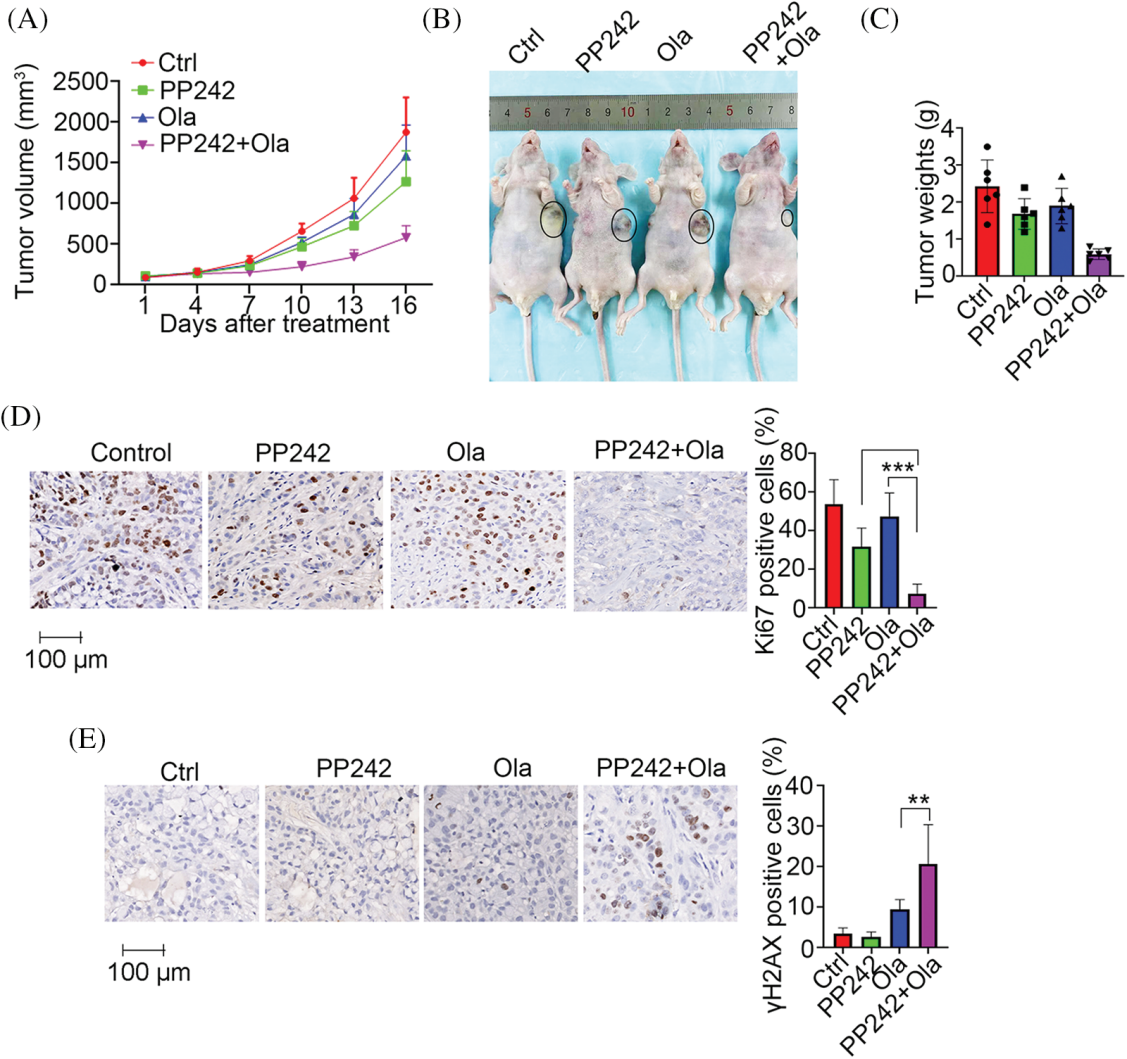
Inhibition of mTORC2 sensitizes PDAC to olaparib treatment. (A–C) Tumor growth curve (A), tumor image (B) and tumor weight graph (C) for PANC-1 xenografts that received control (PBS), PP242 (20 mg/kg), olaparib (100 mg/kg) or both. (D–E) IHC analysis of Ki67 (D) and γH2AX (E) levels in the indicated tumor tissues. Left, representative image of IHC staining; right, quantification of the percentage of positive cells. Data are presented as the mean ± standard deviation (SD), n = 6. **p < 0.01 and ***p < 0.001 according to 2-tailed *t* test.

Since HR-mediated repair capacity dictates PARP inhibitor sensitivity, we hypothesized that inhibiting mTORC2 activity would enhance the PARP inhibitor sensitivity of PDAC cells. We therefore constructed PANC-1 xenografts to evaluate the antitumor efficacy of combination treatment with the PARP inhibitor olaparib and the mTORC2 inhibitor PP242. As expected, the olaparib and PP242 combination synergistically inhibited tumor growth *in vivo* (Figs. 6A–6C). We then performed IHC staining of Ki67, a well-known cell proliferation biomarker, to further confirm the enhanced antitumor efficacy of the olaparib/PP242 combination. Consistent with tumor growth inhibition, the proportion of Ki67-positive cells was dramatically reduced in tumors receiving combination treatment compared with olaparib or PP242 alone (Fig. 6D). As mentioned above, PARP inhibitors kill cancer cells mainly through the generation of DNA DSBs. We thus evaluated γH2AX levels in tumor tissues by IHC analysis. As expected, we observed significantly higher γH2AX levels in olaparib/PP242 combination-treated tumor tissues (Fig. 6E). These results demonstrated that combined treatment with mTORC2 and PARP inhibitors induced synergistic antitumor effects in PDAC cells *in vivo*.

## Discussion

As one of the deadliest cancers, the overall five-year survival rate of pancreatic cancer is approximately 10% [22]. Despite rapid progress in cancer therapeutics, gemcitabine- or fluorouracil-based chemotherapies are still considered the first-line treatments for pancreatic cancer patients [23]. Although chemotherapeutics have improved the survival of pancreatic cancer patients, advances are still limited compared with those in other cancers. Recently, PARP inhibitors have been approved for the treatment of pancreatic cancer patients with BRCA1/2 mutations, which opens a new era of precision medicine for pancreatic cancer. PARP inhibitors, including olaparib, niraparib, veliparib, rucaparib, and talazoparib, have achieved promising therapeutic outcomes and conferred clinically meaningful benefits to pancreatic cancer patients with BRCA1/2 mutations [24]. Recently, study reported that PARP inhibitor pamiparib significantly up-regulated programmed death ligand-1 (PD-L1) expression in pancreatic cancer cells *in vitro* and *in vivo*, consequently, combination of PARPi and PD-1/PD-L1 immune checkpoint inhibitors generated greater therapeutic efficacy in pancreatic cancer patients [25]. Meanwhile, like other targeting therapies, acquired resistance to PARP inhibitors has emerged in clinical management of pancreatic cancer patients. The follow mechanisms would lead to PARP inhibitor resistance: i. Restoration of HR repair capacity in BRCA1/2 mutant cancer cells due to the disruption of DNA end-resection blockers such as 53BP1 and RIF1; ii. Reverse mutations in PARP1, BRCA1, BRCA2; iii. Protection of DNA replication fork from degradation [26,27]. In addition, the overall frequency of BRCA1/2 mutations is only approximately 10% in pancreatic cancer patients [28]. Thus, most pancreatic cancer patients with normal BRCA1/2 function are not eligible to receive PARP inhibitors. In our present study, we showed that mTORC2 signaling is a positive regulator of HR-mediated repair and that targeting mTORC2 kinase significantly decreases HR-mediated repair capacity in pancreatic cancer cells, which consequently increases sensitivity to PARP inhibitors. In terms of the importance of HR-mediated repair in PARP inhibitor sensitivity, our data indicate that mTORC2 is a druggable HR-mediated repair modulator and that mTORC2 inhibition leads to a BRCAness phenotype in cancer cells. In addition, we validated the synergistic antitumor effect of combination treatment with the mTORC2 inhibitor PP242 and the PARP inhibitor olaparib *in vivo*. Thus, our study provides a novel combination strategy to optimize PARP inhibitor treatment in BRCA1/2 wild-type pancreatic cancers.

PDAC progresses rapidly and is resistant to chemotherapies due to its accumulated genetic mutations. Several genetic mutations in tumor driver genes, such as KRAS, EGFR, CDKN2A/p16 and TP53, have been identified to be involved in PDAC development, progression and therapeutic resistance [29,30]. Here, we reported that Rictor, an obligate protein of mTORC2, is overexpressed in PDAC tumor tissues and that knockdown of Rictor greatly inhibited PDAC cell growth and invasion. Therefore, our data imply that hyperactivated mTORC2 is another PDAC driver gene and that targeting its enzymatic activity provides a novel strategy against PDAC.

Given the critical roles of mTOR signaling in maintaining cellular homeostasis, it could affect cancer cell behavior in multiple aspects, including metabolism, energy sensing, and protein synthesis [16,17]. Here, we show that mTORC2 signaling regulates DNA repair by modulating BRCA1 function. mTORC2 positively regulates BRCA1 foci formation, which indicates that mTORC2 plays important roles in BRCA1 localization at DNA DSB sites. However, the underlying mechanism by which mTORC2 directs BRCA1 recruitment to DSBs still needs to be further investigated. In line with previous reports, mTOR and the mTORC2-specific component Rictor are partially localized in the nucleus, where they participate in the regulation of DNA replication and the DNA damage response [31]. Our data showed that mTORC2 regulates DNA repair, which might provide an explanation of the pro-chemoresistance role of mTORC2.

## Data Availability

All the data generated in present study are available from the corresponding author (Jiahua Zhou) upon reasonable request.

## Abbreviations

PDAC: Pancreatic ductal adenocarcinoma
PAAD: Pancreatic adenocarcinoma
PARP: Poly(ADP-ribose) polymerase
mTOR: Mammalian target of rapamycin
mTORC1: mTOR complex 1
mTORC2: mTOR complex 1
HR: Homologous recombination
DSBs: Double-strand breaks
SSB: Single-strand break
WT: Wild-type
HRD: HR repair deficiency
IHC: Immunohistochemistry
IF: Immunofluorescence
TCGA: The Cancer Genome Atlas
IP: Immunoprecipitation
PD-L1: Programmed death ligand-1
